# Considerations for storing and managing retinal images

**Published:** 2023-07-07

**Authors:** Charles Cleland

**Affiliations:** 1 Clinical Research Fellow: International Centre for Eye Health, London School of Hygiene & Tropical Medicine. London, UK.

**Storing and managing retinal images – so that each one is linked to the correct patient – is essential.**

**Figure:**
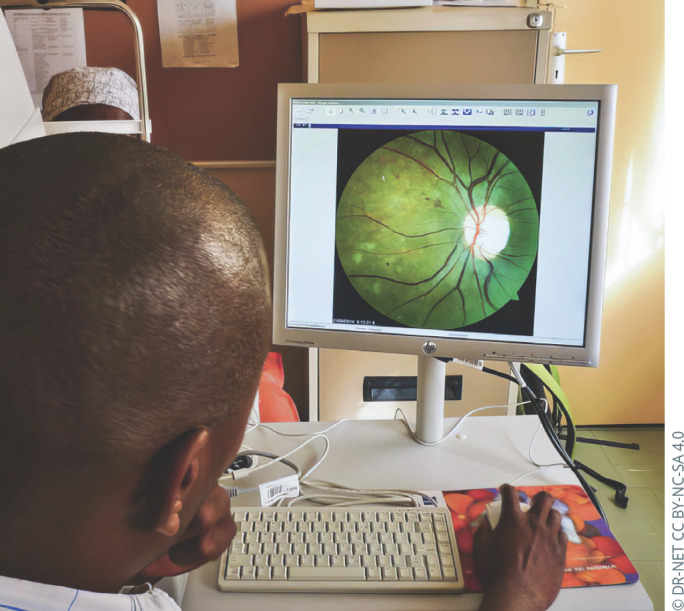
Label image files using a unique patient identifier and the date the image was taken.

The retina is the only tissue in the human body in which blood vessels can be directly visualised. Eye care professionals can therefore use retinal images to detect and diagnose a range of eye conditions, including diabetic retinopathy (DR).

Training screener/graders to take retinal images and grade them has streamlined DR screening, and many more patients living with diabetes are now screened for DR every year. The number of retinal images being taken, stored and exchanged between eye care practitioners and institutions is therefore increasing rapidly.

In a clinical setting, retinal images can be used to:

- Detect DR during screening
- Assess the progression of the disease (by comparing photographs taken during the annual screening visit with those taken the previous year)
- Assess the effects of treatment.

However, for images to be clinically useful, they must be linked to the correct patient information and stored securely and confidentially.

## Labelling image files

Rename all image files using a unique patient identifier (such as a hospital ID) and the date the image was captured. To protect patient confidentiality, any information that could be used to identify the patient (name, date of birth) should be removed from the file name, particularly where the images are not stored in a secure patient database management system.

## Storage and back-up

A majority of retinal cameras have in-built storage capabilities and some have integrated patient database management systems. However, it is important to regularly back up images (ideally monthly) in case a camera malfunctions or is lost/stolen.

The process of backing up images varies between cameras but is straightforward; guidance is provided in manufacturers’ manuals and websites.

There are several options for storage, including using centrally managed hospital servers or cloud storage services (depending on local approval). External hard drives, which have become much less expensive, are an alternative option, but these need to be stored securely, in a locked facility, where only authorised personnel have access.

## How to organise retinal images

The images can be organised and stored by creating individual folders for each patient. The folders can be labelled using a unique patient identifier so a particular patients images can easily be retrieved. Within the folders all the images relating to that particular patient can be stored with the image files labelled using the patient identifier and the date the image was captured.

## Key tips

- Regularly back up images that are stored on cameras, e.g. either in a local server in the hospital if available or using external hard drives
- Link images to clinical information, e.g. by labelling the image file name with a unique patient identifier, such as a hospital ID
- Add the date the image was captured to the image file name
- Remove any personal identifiable information, such as a patient’s name, from the image file name
- Use a filing system that allows you to easily retrieve an individual patient’s images.

**Other reasons to carefully store and manage retinal images**

**Audit and research.** Retinal images can be used for audit or research. For example, a programme may want to explore how effective DR screening has been, or find out which patients are more likely to have advanced disease. There are ethical considerations around this use of patient data, but generally the secondary analysis of anonymised clinical imaging data is acceptable, even if specific consent from the patient (which is often impossible) has not been taken.^1^

**Curation of local data sets for technology development.** There have been many advances in the last few years in the development of artificial intelligence (AI) tools for DR screening. AI systems are trained on large numbers of retinal images that have already been screened by a qualified grader. The AI system ‘learns’ what each DR feature looks like, and is eventually able to grade images based on what it has seen.

The accuracy of an AI system depends on the images it has been shown. If it wasn’t trained on images from specific low- or middle-income countries (LMICs), because of the shortage of local image datasets, the AI may not perform well in those populations. Systematically labelling and storing retinal image data is an important first step in developing retinal image datasets for LMICs. This will help to avoid ’health data poverty’^2^ and reduce inequality in global health.

## References

[1] World Health Organization. Ethics and Governance of artificial intelligence for health: WHO guidance. 2021.

[2] Ibrahim H Liu X Zariffa N Morris AD Denniston AK. Health data poverty: an assailable barrier to equitable digital health care. Lancet Digit Health 2021; 3(4): e260-e5. 33678589 10.1016/S2589-7500(20)30317-4

